# Foods for the Fat

**Published:** 1890-04

**Authors:** 


FOODS FOR THE FAT. A Treatise on Corpulency and a Dietary for its Cure. By Nathaliel E. Davies, member of the Royal College of Surgeons, England. American edition by Chas. W. Greene, M. A., M. D. Philadelphia: J. B. Lippincott Company, 1889. Pp. 132.

This little book is intended for the laity as well as for the physician, and should be read by all. What we eat and how we eat it, as well as how it is prepared, are matters of more consequence than is generally supposed. The plan suggested for reducing fat seems to be a good one. B.

